# Impaired mitochondrial dynamics and removal of the damaged mitochondria in diabetic retinopathy

**DOI:** 10.3389/fendo.2023.1160155

**Published:** 2023-06-21

**Authors:** Kumari Alka, Jay Kumar, Renu A. Kowluru

**Affiliations:** Ophthalmology, Visual and Anatomical Sciences, Wayne State University, Detroit, MI, United States

**Keywords:** diabetic retinopathy, mitochondria, mitofusin, mitophagy, mitochondrial dynamics, retina

## Abstract

**Introduction:**

Mitochondrial dynamic plays a major role in their quality control, and the damaged mitochondrial components are removed by autophagy. In diabetic retinopathy, mitochondrial fusion enzyme, mitofusin 2 (Mfn2), is downregulated and mitochondrial dynamic is disturbed resulting in depolarized and dysfunctional mitochondria. Our aim was to investigate the mechanism of inhibition of Mfn2, and its role in the removal of the damaged mitochondria, in diabetic retinopathy.

**Methods:**

Using human retinal endothelial cells, effect of high glucose (20mM) on the GTPase activity of Mfn2 and its acetylation were determined. Role of Mfn2 in the removal of the damaged mitochondria was confirmed by regulating its acetylation, or by *Mfn2* overexpression, on autophagosomes- autolysosomes formation and the mitophagy flux.

**Results:**

High glucose inhibited GTPase activity and increased acetylation of Mfn2. Inhibition of acetylation, or *Mfn2* overexpression, attenuated decrease in GTPase activity and mitochondrial fragmentation, and increased the removal of the damaged mitochondria. Similar phenomenon was observed in diabetic mice; overexpression of *sirtuin 1* (a deacetylase) ameliorated diabetes-induced inhibition of retinal Mfn2 and facilitated the removal of the damaged mitochondria.

**Conclusions:**

Acetylation of Mfn2 has dual roles in mitochondrial homeostasis in diabetic retinopathy, it inhibits GTPase activity of Mfn2 and increases mitochondrial fragmentation, and also impairs removal of the damaged mitochondria. Thus, protecting Mfn2 activity should maintain mitochondrial homeostasis and inhibit the development/progression of diabetic retinopathy.

## Introduction

Diabetic retinopathy remains the leading cause of vision loss in the working age adults, and the exact molecular mechanism of this devastating disease remains unclear. Constant chronic exposure to high circulating glucose damages mitochondria and increased production of mitochondrial free radicals affects major metabolic abnormalities associated with the development of diabetic retinopathy (1–3). Mitochondria become swollen with partial or total cristolysis, their membrane potential is impaired and cytochrome c leaks out in the cytosol, activating the apoptotic machinery. Mitochondrial biogenesis and copy numbers are decreased and their DNA (mtDNA) is damaged, and continuous production of free radicals from the compromised electron transport chain system continues to self-propagate; increased production of mitochondrial free radicals is implicated in the major metabolic abnormalities associated with the development of diabetic retinopathy (2–6).

Mitochondria are highly dynamic in nature, and depending on the energy demand, they can alter their shape through fusion and fission events (7, 8). The balance between fission and fusion, ‘mitochondrial dynamics’, regulates their size, number, distribution and quality control in cells. While fusion allows mitochondria to integrate the contents of two mitochondria diluting the damaged contents, fission is linked to impairments in mitochondrial respiration, membrane permeability and in the segregation of the damaged mitochondrial components for degradation by autophagy (9, 10). Fission and fusion are regulated by small GTPases; dynamin-related protein-1 (Drp1) helps in fission, mitofusin 1 and 2 (Mfn1 and Mfn2 respectively) control outer mitochondrial membrane fusion and Optic atrophy-1 facilitates inner membrane fusion (11, 12). In diabetic retinopathy, fusion-fission process is dysregulated, while Drp1 is upregulated, Mfn2 is downregulated in retinal vascular cells, and the mitochondria are fragmented (13–15).

Posttranslational modifications are intimately implicated in enzyme regulation as they can either activate an enzyme, or inhibit it. Proteins associated with mitochondrial dynamics undergo posttranslational modifications including acetylation and ubiquitination; Mfn2, a key player in mitochondrial fusion, is shown to undergo many posttranslational modifications (16), and acetylation is considered as one of the major posttranslational modifications implicated in the regulation of its activity (17, 18). Sirutin1 (Sirt1) is one of the deacetylating enzymes responsible for maintaining acetylation status of proteins, and in diabetes, its expression and activity are decreased in the retina and its capillary cells (19). The role of acetylation in the regulation of GTPase activity of Mfn2 in diabetic retinopathy is unclear.

Impaired fusion process is associated with the generation of small, depolarized and dysfunctional mitochondria, and in order to maintain mitochondrial homeostasis, the damaged mitochondria have to be removed. Mitochondria are engulfed by autophagosomes, and a cytosolic protein LC3-phosphatidylethanolamine conjugate (LC3B) is recruited to the membranes of the autophagosomes, facilitating the fusion of autophagosomes with the lysosomes to degrade the intracellular components (20–22). Mfn2, by increasing autophagosome formation and fusion of autophagosome-lysosome, plays an important role in the mitophagy process (8, 23, 24). Whether Mfn2 has any role in the removal of the damaged mitochondria in the development of diabetic retinopathy is not understood.

Our aim was to investigate the mechanism responsible for inhibition of Mfn2, and the role of Mfn2 in the removal of the damaged mitochondria, in diabetic retinopathy. Using human retinal endothelial cells (HRECs), we have investigated the effect of high glucose on the GTPase activity of Mfn2 and its acetylation status, and the role of Mfn2 in the removal of damaged mitochondria. Key results are confirmed in the retina from streptozotocin-induced diabetic mice overexpressing *Sirt1*.

## Methods

### *In vitro* model

HRECs from Cell Systems Corporation (Cat. no. ACBRI 181, Cell Systems Corp, Kirkland, WA, USA) were cultured in an environment of 95% O_2_ and 5% CO_2_ in Dulbecco’s modified Eagle medium (DMEM, Cat. no. D5523; Sigma-Aldrich Corp., St. Louis, MO, USA) supplemented with 20μg/ml endothelial cell growth supplement, 12% heat-inactivated fetal bovine serum and 1% each insulin- transferrin- selenium-Glutamax and antibiotic/antimycotic. HRECs from the 6^th^-8th passage were incubated in 5mM D-glucose (NG) or 20mM D-glucose (HG) for 96 hours, in the presence or absence of a Sirt1 activator, 25μM resveratrol (25) (Cat. no. CAS 42206-94-0, Sigma-Aldrich). Each experiment had an osmotic control where HRECs were incubated in 20 mM L-glucose (L-Gl), instead of 20mM D-glucose. A group of HRECs from 5^th^-6^th^ passage were transfected with *Mfn2* overexpressing plasmids using Turbofectin reagent (Cat. No. RG202218 and TF81001; OriGene, Rockville, USA), as described previously (13). After incubating for eight hours with the plasmids, cells were washed with DMEM and incubated in either normal or high glucose for 96 hours. Parallel transfection with the empty vector was run in each experiment. The transfection efficiency of *Mfn2* was determined by quantifying its gene transcripts by quantitative real-time PCR (qRT-PCR).

### *In vivo* model

Mice (~20g body weight), wild-type C57BL/6J or overexpressing *Sirt1* (*St, C57BL/6-Actbtm3.1 (Sirt1) Npa/J*), were made diabetic by streptozotocin injection (55 mg/kg BW) for 4 consecutive days. Three days after the last injection of streptozotocin, their blood glucose was measured, and mice with >250 mg/dl blood glucose were considered diabetic. Each group had 10-12 mice with equal numbers of males and females (19). The average blood glucose values were not different in male and female mice (~450mg/dl).

Mice were sacrificed six months after induction of diabetes by carbon dioxide inhalation, and their retina was isolated immediately under a dissecting microscope (19). The retina was homogenized in RIPA buffer (50mM Tris-HCl, pH 8.0, 150 mM NaCl, 1% NP40, 0.05% sodium deoxycholate, 0.1% SDS) containing protease inhibitors. The homogenate was centrifuged at 10,000Xg for 10 minutes, and the lysate was used for measuring GTPase activity of Mfn2.

The treatment of animals was in accordance with the guidelines of the Association for Research in Vision and Ophthalmology Resolution on the Use of Animals in Research, and the experimental protocols were approved by Wayne State University’s Animal Care and Use Committee. This study is reported in accordance with the ‘Animal research: reporting of *in vivo* experiments (ARRIVE) guidelines.

### Gene expression

Gene transcripts of *Mfn2* were quantified in the Trizol-isolated RNA by SYBR green-based qRT-PCR using *Mfn2* primers (Fwd- ATGCAGACGGAAAAGCACTT, Rev- ACAACGCTCCATGTGCTGCC), and β-actin was employed as a housekeeping gene (Fwd- AGCCTCGCCTTTGCCGATCCG, Rev- TCTCTTGCTCTGGGCCTCGTCG) (13).

GTPase activity of Mfn2 was measured using the GTPase assay kit (DATG-200; Bioassay Systems, Hayward, CA, USA), similar to the one recently used for the GTPase activity of the mitochondrial fission protein Drp1 (15). HRECs/retina were homogenized in the RIPA buffer and Mfn2 was immunoprecipitated from 100μg lysate using 3μg Mfn2 monoclonal antibody (Cat. No. ab56889, Abcam, Cambridge, MA). The pellet was incubated with Protein A/G Plus agarose beads suspended in the lysis buffer and the beads were washed, and incubated with 0.5mM GTP for 30 minutes at room temperature. The phosphate released was quantified spectrophotometrically at 620 nm. Percentage change in the phosphate released was calculated considering the values obtained from HRECs in normal glucose, or wildtype normal mouse, as 100%.

Acetylation of Mfn2 was determined by immunofluorescence microscopy using Mfn2 and acetyl lysine primary antibodies (Cat. No. ab56889 and Cat. No. ab21623, respectively, Abcam; 1:250 dilution each). Their secondary antibodies included Alexa Fluor-488 (green) conjugated anti-rabbit (Cat. No. A11000, Molecular Probes-Life Technologies, Grand Island, NE, USA; 1:500 dilution) and Texas red-conjugated anti-mouse (Cat. No. TI-2000 Vector Laboratories, Burlingame, CA, USA; 1:500 dilution), respectively. Immuno-labelled cells were mounted using DAPI-containing Vectashield mounting medium (Cat. no. H-1000 Vector Laboratories), and were examined under an ApoTome microscope (Carl Zeiss, Chicago, IL, USA) with a 20X objective. Fluorescence intensity of acetyl lysine was quantified using Zeiss software module and the data are expressed as arbitrary units (AU). Pearson correlation coefficient between acetyl lysine and Mfn2 was calculated using Zeiss software module (15, 26).

Acetylation of Mfn2 was confirmed by coimmunoprecipitation technique, as described previously (19, 27). Protein (100-150μg) was incubated overnight at 4°C with 3μg acetyl lysine antibody (Cat. No. 9441, Cell Signaling Technology, Inc., Beverly, MA, USA), followed by incubation of the immunoprecipitated pellet with pre-washed beads suspended in the lysis buffer (Protein A and G Plus; Cell Signaling Technology, Inc.). Proteins were separated on SDS-PAGE, and the membranes were immunoblotted for Mfn2.

Mitochondrial fragmentation was performed by live cell imaging in HRECs (on coverslips) incubated in normal glucose or in high glucose with or without resveratrol. Cells were incubated with 200nM MitoTracker green FM (Cat. No. M7514, Thermo Fisher Scientific, Waltham, MA, USA) for 10 minutes, and after washing the coverslips with PBS (3X), they were imaged under Zeiss ApoTome fluorescence microscope using a 40X objective (13, 28).

Mitochondrial membrane potential was measured using, JC-1 (Cat. No. MP03168, Molecular Probes, Carlsbad, CA, USA), a cationic dye which enters into the healthy mitochondria forming red fluorescent J-aggregates, as reported previously (29, 30). At the termination of the experimental incubations, HRECs were washed with PBS and incubated with 5µM JC-1 in DMEM for 30 minutes at 37°C. Excess dye was removed by washing the coverslips with PBS, and the cells were visualized under Zeiss ApoTome at 20X objective. Fluorescence intensities of the original green monomers (485 nm excitation and 530 nm emission) and the red aggregates (525 nm excitation and 590 nm emission) were measured using Zeiss software module, and the ratio of red and green fluorescence was plotted.

Autophagosomes formation in HRECs was assessed using an Autophagy Detection kit (Cat. no. ab139484; Abcam), according to the manufacturer’s protocol. In brief, after experimental incubation, the cells (in a 96-well plate) were incubated with the detection reagent at 37°C for 30 minutes. Fluorescence was measured in a microplate reader at 480nm excitation and 530nm emission wavelengths (31).

Autophagosomes formation in the mouse retina was quantified using an ATG12 mouse ELISA Autophagy Detection kit (Cat. no. MBS2706904; Mybiosource, San Diego, CA,USA), following manufacturer’s instructions. In brief, retinal lysate (25µg protein) was incubated in the ELISA plate for one hour at 37°C. After aspirating, the plate was incubated at 37°C with the detection reagent A for one hour, followed by with reagent B for 30 minutes. The plate was then incubated with the substrate for 20 minutes, and the reaction was terminated by the addition of the stop solution. ATG12 was measured spectrophotometrically at 450nm wavelength.

LC3B recruitment in the mitochondria was quantified by immunofluorescence microscopy using LC3B antibody (Cat. no. PA1-46286, Invitrogen;1:500) and Cox IV as a mitochondrial marker (Cat. No. sc-376731, Santa Cruz Biotechnology; 1:500). While Texas red-conjugated secondary antibody (Cat. No.TI200, Vector Laboratories, Burlingame, CA, 1:1000 dilution) was used for LC3B, a DyLight green-conjugated secondary antibody (Cat. no. ab201799, Abcam; 1:1000 dilution) was used for CoxIV.

Mitochondria-lysosome fusion was determined by fluorescence microscopy by incubating HRECs for 30 minutes at 37°C in the dark with 200nM LysoTracker (an acidotropic fluorescent probe which labels and tracks acidic organelles in the living cells, (Cat. no. L7528, Thermo Fisher Scientific) (32) and 200nM MitoTracker green (Cat. No. M7514, Thermo Fisher Scientific), for labeling the lysosomes and the mitochondria, respectively. The coverslips were washed with PBS (3X), and Live cell images were captured using a 40X objective (33). The fluorescence intensity of LysoTracker and Pearson correlation coefficient between LysoTracker and MitoTracker were determined using Zeiss software module.

Mitophagy flux was detected using the Mitophagy detection kit (Cat. No. MD01-10, Dojindo Molecular Technologies, Rockville, USA). Briefly, after the desired treatments, cells were washed with DMEM and incubated with 100nM Mtphagy Dye (diluted in DMEM) for 30 min at 37°C. This was followed by washing the cells with DMEM, and trypsinizing them. After removing the trypsin by washing cells (2x) with the FACS buffer (0.5% BSA in PBS), fluorescence intensity of Mtphagy Dye was quantified by flow cytometry under PerCP Cy5.5 channel (excitation/emission= 486/679). Raw Flow Cytometry Standard files were analyzed by FlowJo v10.8.1 software (BD Biosciences, San Jose, CA, USA). Relative Mtphagy dye scattering, proportional to the amount of mitophagy (34), was plotted.

Mitophagy in mouse retina was determined by incubating small pieces of the retina with 50µl of Accumax™ (Cat No. A7089, Sigma-Aldrich Corp.) for 10 minutes at 37°C. After washing the digested tissue with DMEM containing 10% fetal bovine serum, and filtering through a 40μm cell strainer (35), cell suspension was incubated with 25nM MitoTracker deep red (Cat No. M22426, Thermo Fisher Scientific) for 30 minutes at 37°C. Cells were washed with the flow buffer (0.5% BSA in PBS) three times and scanned under FL3 640nm wavelength in the flow cytometer, and the raw Flow Cytometry Standard files were analyzed by FlowJo v10.8.1 software.

Statistical analysis: Data are presented as mean ± standard deviation. Comparison between groups was made using one-way ANOVA, followed by ‘Dunn *post hoc*’ test, and a p<0.05 was considered significant.

## Results

**Figure 1:**
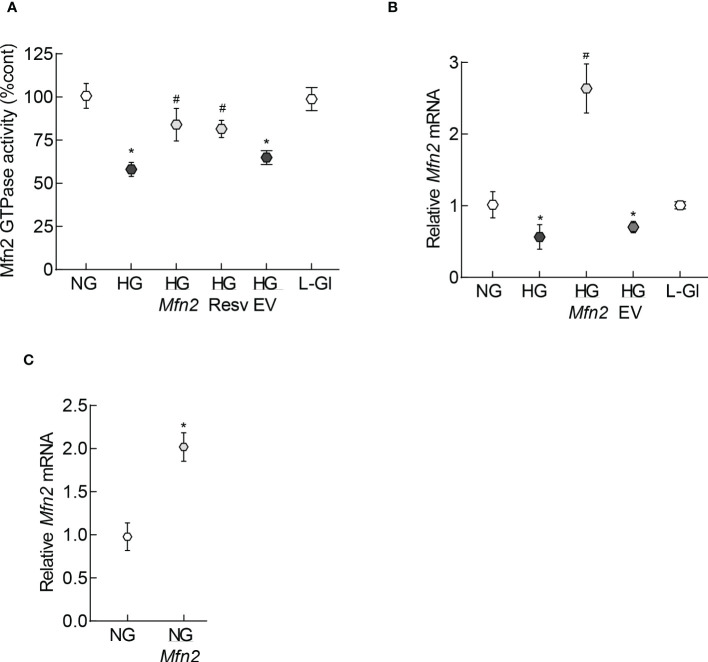
GTPase activity of Mfn2. **(A)** GTPase activity of Mfn2 was measured by quantifying the release of phosphate spectrophotometrically at 620 nm. Percentage change in the phosphate released was calculated based on the values obtained from cells in normal glucose as 100%. **(B)** Gene transcripts of *Mfn2* were quantified by qRT-PCR using *β-actin* as a housekeeping gene. **(C)** Transcription efficiency of *Mfn2* was determined by quantifying its RNA in the cells before incubating in any experimental condition. Each experiment was performed in triplicate in three or more different cell preparations, and the values are represented as mean ± SD. NG= 5mM D-glucose, HG= 20mM D-glucose, HG/Mfn2 and HG/EV= cells transfected with *Mfn2* overexpressing plasmids or empty vector, and incubated in 20mM D-glucose, respectively; L-Gl= 20mM L-glucose; NG/Mfn2= cells transfected with *Mfn2* overexpressing plasmids in normal glucose, before initiating any experimental incubation.*p<0.05 vs NG and ^#^<0.05 vs HG.

Mitochondrial fragmentation is increased and the expression of Mfn2 is downregulated in retinal capillary cells and in hyperglycemia (13). To understand how diabetes increases fragmentation, GTPase activity of Mfn2 was quantified. As shown in **Figure 1A**, GTPase activity was reduced by over 40% in HRECs incubated in high glucose (20mM D-glucose, HG group), compared to normal glucose (5mM D-glucose, NG group), and overexpression of *Mfn2* ameliorated glucose-induced decrease in the GTPase activity. Values obtained from cells untransfected or transfected with *Mfn2* overexpressing plasmids, and incubated in high glucose, were significantly different from each other (p<0.01). However, GTPase activity of Mfn2 in untransfected cells or cells transfected with the empty vector (EV), and incubated in high glucose, wase not different from each other (p>0.01). Consistent with the GTPase activity, in the same cell preparation, glucose-induced decrease in *Mfn2* gene expression was also protected by *Mfn2* overexpression (**Figure 1B**). **Figure 1C** is included to show the transfection efficiency of *Mfn2*; *Mfn2* gene transcripts were about two-fold higher in *Mfn2* overexpressing cells, compared to untransfected cells.

**Figure 2:**
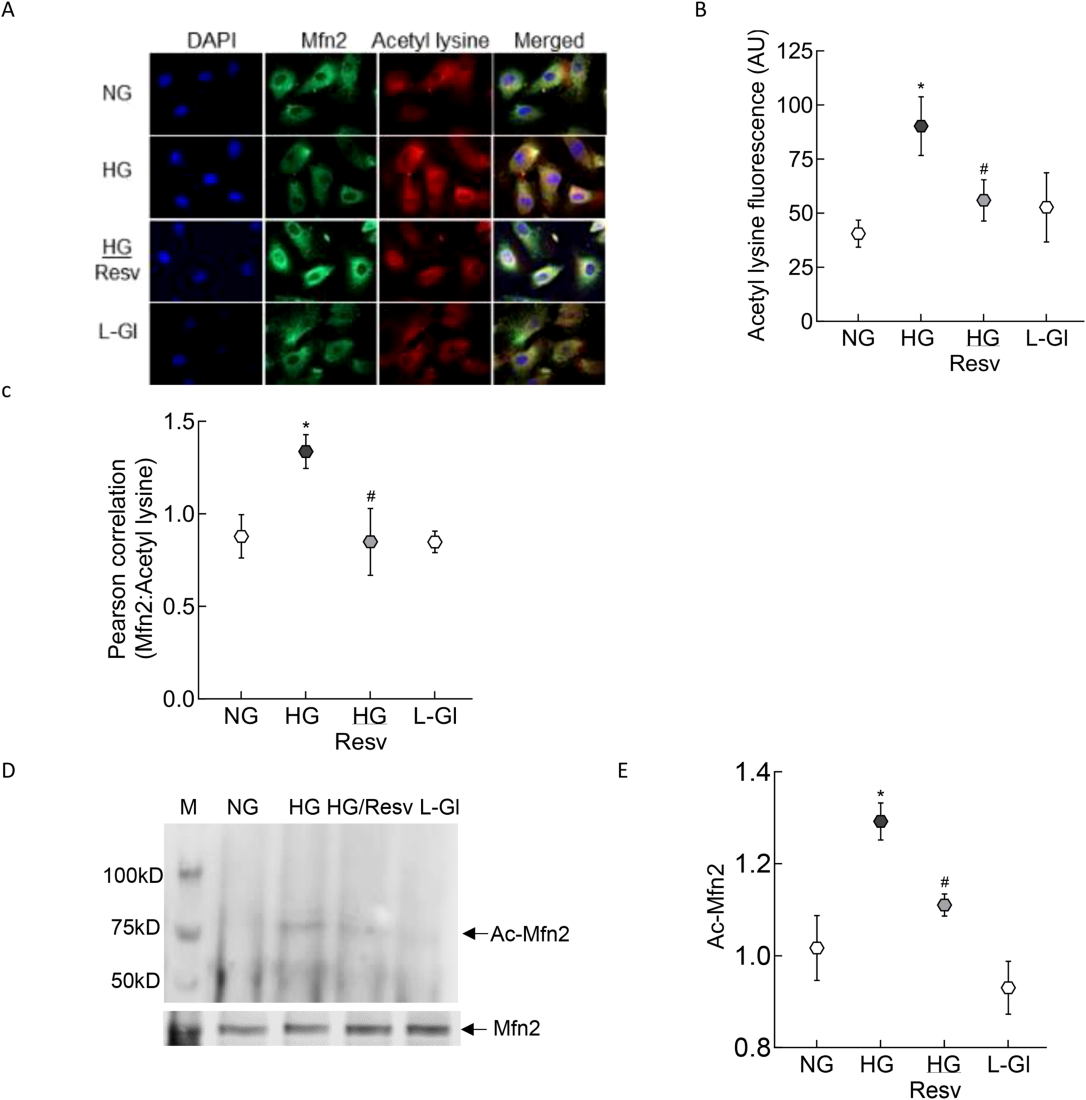
Acetylation of Mfn2. **(A)** Representative immunofluorescence image showing staining of Mfn2 (Alexa Fluor-488 conjugated secondary antibody, green) and acetyl lysine (Texas red-conjugated secondary antibody). Cells were imaged under an ApoTome microscope with a 20X objective. **(B)** Fluorescence intensity of acetyl lysine was quantified using Zeiss software module and the data are expressed as arbitrary units (AU). **(C)** Pearson correlation coefficient between acetyl lysine and Mfn2 was calculated using Zeiss software module. **(D)** Representative image of Mfn2 western blot in acetyl lysine immunoprecipitated HRECs. **(E)** Intensity of the acetylated Mfn2 (Ac-Mfn2) was quantified by image J software. The values are represented as mean ± SD. NG= 5mM D-glucose, HG= 20mM D-glucose, HG/Resv = cells incubated in 20mM D-glucose in the presence of resveratrol; L-Gl= 20mM L-glucose.*p<0.05 vs NG and ^#^p<0.05 vs HG.

To examine the mechanism responsible for inhibition of Mfn2 GTPase activity, its acetylation status was determined; fluorescence intensity of acetyl lysine was significantly higher in HRECs incubated in high glucose compared to normal glucose (**Figures 2A, B**). Consistent with this, Pearson correlation coefficient of Mfn2 and acetyl lysine, quantified from 25-30 cells/group, was also significantly higher in the cells in high glucose compared to normal glucose or 20mM L-glucose (p<0.01; **Figures 2C**). However, fluorescence intensity of acetyl lysine, obtained from cells in high glucose in the presence of a deacetylase activator, resveratrol (HG/Resv group), was not different from that obtained from cells in normal glucose or 20mM L-glucose (p>0.01). Acetylation status of Mfn2 was also confirmed by co-immunoprecipitation technique, and as shown in **Figures 2D, E**, high glucose incubated cells had higher acetylated Mfn2 compared to cells in normal glucose or 20mM L-glucose.

**Figure 3:**
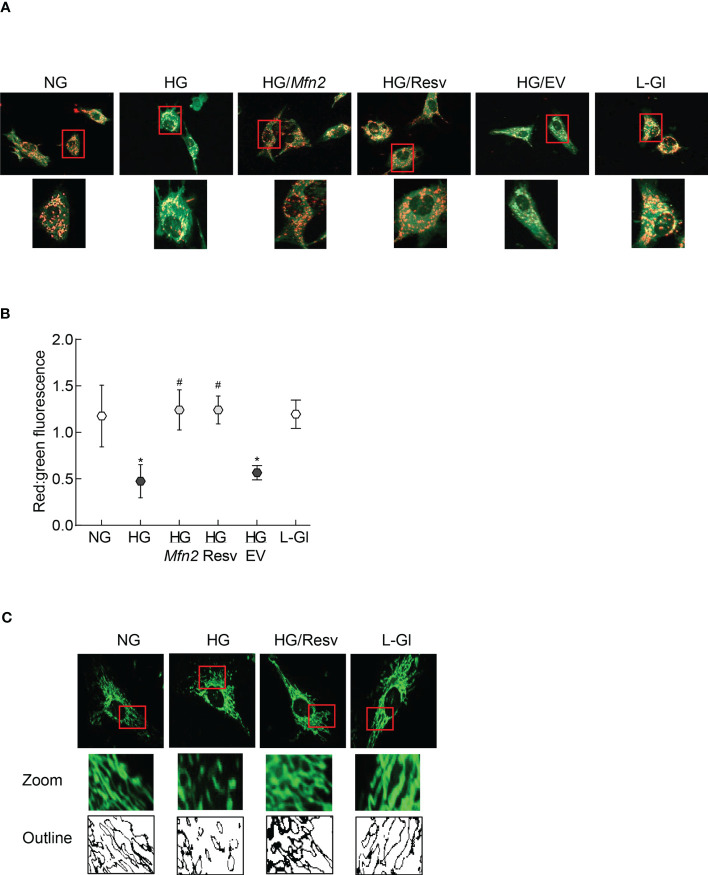
Regulation of Mfn2 on mitochondrial membrane potential and fragmentation. **(A)** Representative image using cationic dye JC-1 showing red fluorescent J-aggregates and green J-monomers. **(B)** Graph showing red and green fluorescence intensity ratio. Values are represented as mean ± SD from 3-4 slides/group, done in three cell preparations. **(C)** Image of mitochondrial fragmentation in the live cells using MitoTracker green dye, imaged under an ApoTome microscope using a 40X objective. The area inside the red box is enlarged, and bare outlines are drawn to show the morphology. NG and HG= 5mM and 20mM D-glucose; HG/Mfn2 and HG/EV= cells transfected with *Mfn2* overexpressing plasmids or empty vector, and incubated in 20mM D-glucose, respectively; HG/Resv = 20mM D-glucose + resveratrol L-Gl= 20mM L-glucose.*p<0.05 vs NG and ^#^p<0.05 vs HG.

The role of Mfn2 and its acetylation in mitochondrial integrity was examined by quantifying mitochondrial membrane potential. As reported previously, compared to normal glucose, fluorescence was mainly original green (monomers) in the cells in high glucose with very few red fluorescent J-aggregates, and the ratio of red to green fluorescence intensity was significantly lower. *Mfn2* overexpressing cells in high glucose (HG/*Mfn2* group) had higher number of red fluorescent J-aggregates compared to the empty vector transfected cells in high glucose (HG/EV group), and the ratio of red to green fluorescence intensity in HG/*Mfn2* group was similar to that obtained from the cells in normal glucose or 20mM L-glucose. Furthermore, resveratrol supplemented, high glucose incubated cells (HG/Res group) had higher number of red fluorescent J-aggregates, and red to green fluorescence ratio was not different from that obtained from cells in normal glucose (**Figures 3A, B**). Consistent with ameliorating changes in mitochondrial membrane potential, resveratrol also attenuated glucose-induced increased mitochondrial fragmentation (**Figure 3C**).

**Figure 4:**
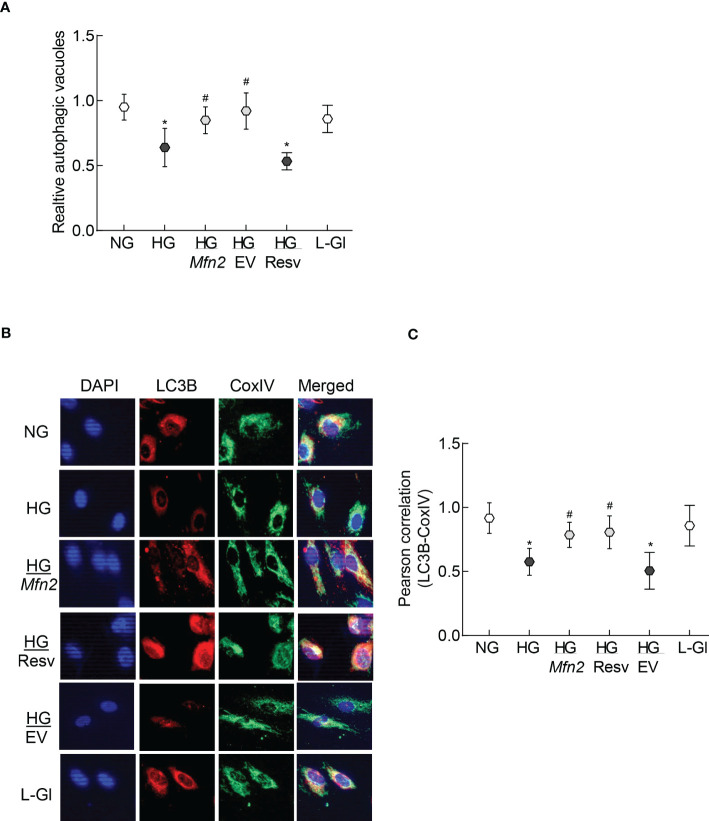
Autophagosomes formation and LC3B recruitment in the mitochondria. **(A)** Relative autophagic vacuoles were measured using a cationic amphiphilic tracer dye. **(B)** A representative image of dual staining of HRECs with LC3B and CoxIV antibodies using Texas red-conjugated and a DyLight green-conjugated secondary antibodies, respectively, and **(C)** Pearson correlation showing the coefficient of interaction between LC3B and CoxIV from 25-30 cells in each group. Values in the graphs are represented as mean ± SD. NG and HG= 5mM and 20mM D-glucose; HG/Mfn2 and HG/EV= cells transfected with Mfn2 overexpressing plasmids or empty vector, and incubated in 20mM D-glucose, respectively; HG/Resv = 20mM D-glucose + resveratrol L-Gl= 20mM L-glucose.*p<0.05 vs NG and ^#^p<0.05 vs HG.

Removal of damaged mitochondria requires the formation of autophagosome, followed by fusion of the autophagosome with the lysosome and degradation of the damaged mitochondria (36), and Mfn2 is also considered to play a role in the removal of the damaged mitochondria (24). The role of Mfn2 in the removal of damaged mitochondria was investigated by first determining the effect of *Mfn2* overexpression in the autophagosome formation. As shown in **Figure 4A**, relative number of fluorescent autophagic vacuoles was decreased significantly in the cells in high glucose, compared to normal glucose (p>0.05), and this decrease was ameliorated by overexpression of *Mfn2* (HG/*Mfn2* group), or by the addition of resveratrol (HG/Resv group). Damaged mitochondria are engulfed by autophagosomes, and the recruitment of LC3B in the membranes of the autophagosomes allows them to fuse with the lysosomes for degradation. To further investigate the role of Mfn2 in the removal of the damaged mitochondria, effect of *Mfn2* overexpression on LC3B recruitment at the mitochondria was investigated. Co-staining of LC3B and mitochondrial marker, CoxIV, was reduced significantly in the cells in high glucose vs normal glucose, which was ameliorated in *Mfn2* overexpressing cells (**Figure 4B**). Compared to cell in normal glucose, Pearson-correlation coefficient of LC3B and CoxIV was significantly decreased in HG group, but HG/*Mfn2* and HG/Resv groups had similar Pearson-correlation coefficients, and the values were significantly higher than those obtained from cells in HG or HG/EV groups (**Figure 4C**).

**Figure 5:**
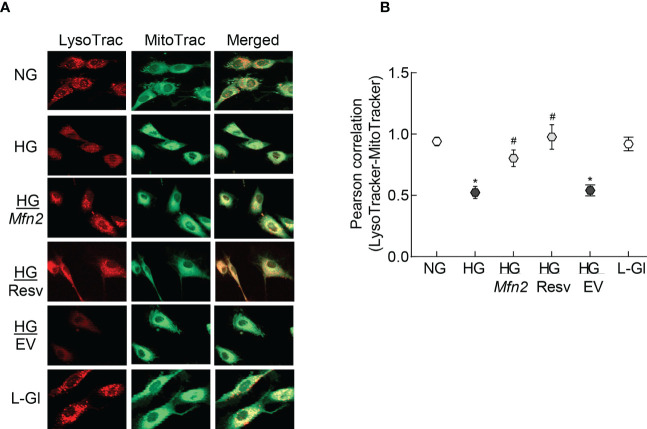
Mitochondria-lysosomes fusion. **(A)** HRECs were stained with LysoTracker and MitoTracker, and live cell images were captured in ApoTome using a 40X objective. **(B)** Pearson correlation coefficient between LysoTracker and MitoTracker was calculated in 2-3 slides/group, each repeated with 3 different HREC preparations (total of 30-40 cells/group). NG= 5mM D-glucose; HG=20mM D-glucose; HG/*Mfn2* and HG/EV= cells transfected with *Mfn2* overexpressing plasmids or empty vector, and incubated in 20mM D-glucose, respectively; HG/Resv = 20mM D-glucose in the presence of resveratrol L-Gl= 20mM L-glucose.*p<0.05 vs NG and ^#^p<0.05 vs HG.

To examine mitochondrial entrapment inside the autolysosomes, co-labeling with LysoTracker and MitoTracker was performed. Compared to normal glucose, cells in high glucose had decreased co-labeling of LysoTracker and MitoTracker (**Figure 5A**), which was further confirmed by lower LysoTracker-MitoTracker Pearson correlation coefficient (**Figure 5B**), suggesting poor removal of the damaged mitochondria. Overexpression with *Mfn2* or resveratrol supplementation (HG/*Mfn2* and HG/Resv groups, respectively) prevented glucose-induced decrease in LysoTracker-MitoTracker co-staining and their Pearson correlation coefficient.

**Figure 6:**
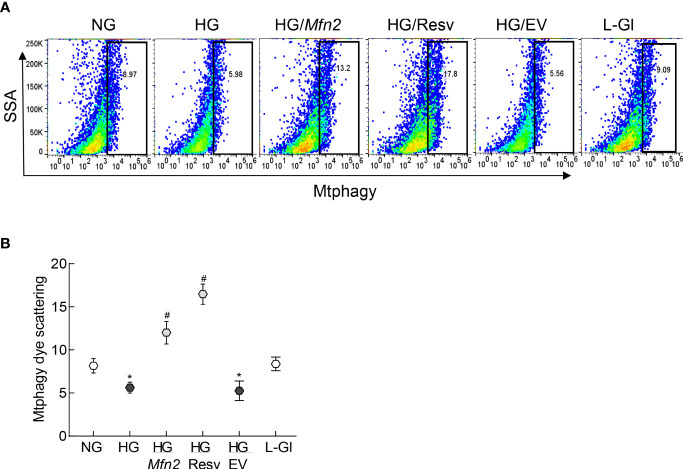
Regulation of Mfn2 and mitophagy flux. **(A)** Cells stained with Mtphagy Dye were scanned under PerCP Cy5.5 channel in a BD Accuri C6 plus flow cytometer, and the data analyzed by FlowJo v10.8.1 software. **(B)** Plot showing relative scattering of Mtphagy dye in various groups; values represented as mean ± SD. NG and HG= 5mM and 20mM D-glucose; HG/Mfn2 and HG/EV= cells transfected with *Mfn2* overexpressing plasmids or empty vector, and incubated in 20mM D-glucose, respectively; HG/Resv = 20mM D-glucose + resveratrol L-Gl= 20mM L-glucose.*p<0.05 vs NG and ^#^p<0.05 vs HG.

Consistent with autophagosome-autolysosomes formation, cells in high glucose had reduced scattering of the Mtphagy dye compared to normal glucose, indicating a decreased mitophagy flux and poor removal of the damaged mitochondria. However, *Mfn2* overexpression, or supplementation with resveratrol, prevented this glucose-induced decrease in scattering (**Figures 6A, B**). Values obtained from cells untransfected, or transfected with empty vector, and incubated in high glucose, were not different from each other, but were significantly different from those obtained from untransfected cells in normal glucose or in 20mM L-glucose.

**Figure 7:**
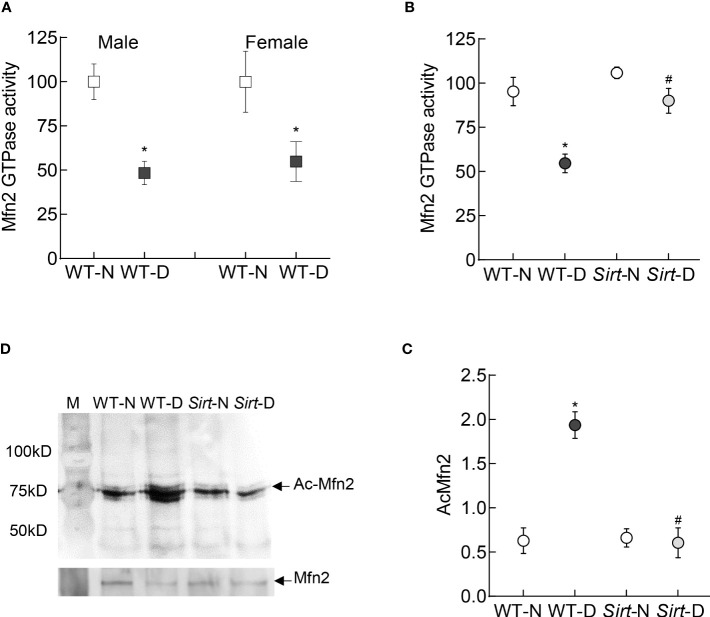
Retinal Mfn2 GTPase activity and acetylation in diabetic mice. Retinal Mfn2 activity was measured spectrophotometrically by quantifying the release of phosphate from the conversion of GTP to GDP in **(A)** WT male and female mice, and **(B)** *Sirt1* overexpressing mice maintained normal or diabetic. Values obtained from wildtype normal control mice were considered as 100%. **(C)** Representative Mfn2 western blot in acetyl lysine immunoprecipitated retinal samples and **(D)** relative acetylated Mfn2 (Ac-Mfn2), quantified by image J software. Values are represented as mean ± SD obtained from 4-6 mice in each group, each measurement made in duplicate. WT-N and WT-D= wild type normal and diabetic mice; *Sirt*-N and *Sirt*-D= *Sirt1* overexpressing mice maintained normal or diabetic.*p<0.05 vs WT-N and ^#^p<0.05 vs WT-D.

In accordance with the *in vitro* results, the GTPase activity of Mfn2 was decreased by ~50% in the retina of wild type diabetic mice (WT-D) vs wild type normal mice (WT-N) in both male and female mice (**Figure 7A**). Please note that the results presented for rest of the experiments are pooled from both male and female mice. However, overexpression of *Sirt1* attenuated diabetes-induced decrease in Mfn2 activity (**Figure 7B**). Although Mfn2 activity in *Sirt1* overexpressing diabetic mice (*Sirt*-D) was slightly lower than that in normal mice (*Sirt*-N), it was not significantly different from that obtained from mice in WT-N and *Sirt*-N groups (p>0.05), instead was significantly different from that obtained from mice in WT-D group (p<0.05). Consistent with decrease in the GTPase activity, Mfn2 acetylation was higher in mice in WT-D group, compared to WT-N group. However, Mfn2 acetylation in *Sirt*-N, *Sirt*-D groups was similar to that in WT-N group, and was also significantly lower than in WT-D group (p<0.01; **Figures 7C, D**).

**Figure 8:**
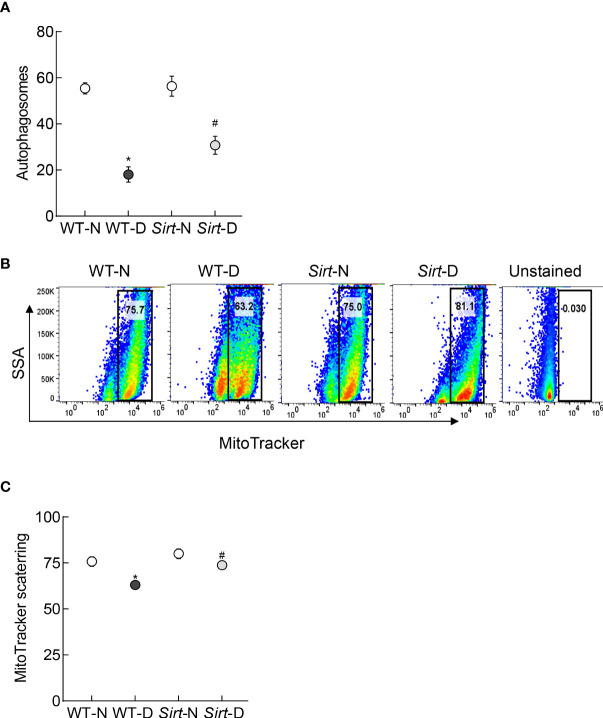
Effect of *Sirt1* overexpression on retinal mitophagy. **(A)** ATG12 levels were quantified by an ELISA method, and **(B)** mitophagy flux was quantified in retinal cell suspension by Flow Cytometry using MitoTracker deep red. **(C)** Representation of relative scattering of MitoTracker red. Each measurement was made in 5-6 mice/group. WT-N and WT-D= wild type normal and diabetic mice; *Sirt-*N and *Sirt*-D= Sirt1 overexpressing mice. *p<0.05 vs WT-N and ^#^p<0.05 vs WT-D.

Autophagosome formation and mitophagy flux were decreased significantly in the retina from mice in WT-D group, compared to WT-N group, and *Sirt1* overexpression ameliorated diabetes-induced decrease in autophagosomes and mitophagy flux. The values in *Sirt*-D were not different from the values in WT-N and *Sirt*-N groups (p>0.01), but were significantly higher compared to values in WT-D (p>0.05; **Figure 8**).

## Discussion

Mitochondria are highly dynamic organelles, and the equilibrium between mitochondrial fusion and fission events are crucial for many cellular processes including mitochondrial quality control and apoptosis (7, 8). In diabetes, mitochondrial dynamic is imbalanced; while mitochondrial fusion is decreased, fission is increased, and dysfunctional mitochondria result in accelerated retinal capillary cell apoptosis, a phenomenon which precedes the development of diabetic retinopathy (13–15). Mitochondrial fusion and fission are mediated in a GTPase-dependent manner (11, 12). In diabetes, the expression (protein and gene) of Mfn2, one of the major proteins associated with the fusion of outer membrane, is downregulated, and mitochondrial fragmentation is increased (13, 14). Here, our results show that the GTPase activity of Mfn2 is decreased and its acetylation is increased in the retina and its endothelial cells in hyperglycemic milieu. Regulation of acetylation prevents hyperglycemia-induced reduction in the GTPase activity of Mfn2 and increase in mitochondrial fragmentation. Furthermore, we show that overexpression of *Mfn2*, or inhibition of its acetylation, also improves the removal of the damaged mitochondria. Similar phenomenon is observed in the retina of diabetic mice where overexpression of *Sirt1* ameliorates diabetes-induced decrease in the GTPase activity of Mfn2, and the formation of autophagosomes for the removal of the damaged mitochondria. These results suggest that acetylation plays a major role in maintaining mitochondrial dynamics by regulating the GTPase activity of Mfn2, and inhibition of Sirt1 in diabetes reduces its GTPase activity, resulting in increased mitochondrial fragmentation. Subnormal Mfn2 activity further worsen the cellular metabolism by impairing the removal of the damaged mitochondria.

Mitochondrial dynamics requires a balance between fusion and fission; the fusion of the double membraned mitochondria is a complex process which involves the mitochondria to first physically and reversibly tether. This is followed by fusion of the outer membranes in a GTPase dependent manner, and then the fusion of inner membranes. Among the two isoforms of mitofusin responsible for outer membrane fusion, Mfn2 is also implicated in many other mitochondrial activities including trafficking, turnover and apoptosis, and its GTPase activity serves as an initial mitochondrial anchor by dimerizing during GTP hydrolysis (37, 38). Mfn2 expression is decreased in hyperglycemic milieu, and its overexpression prevents glucose-induced mitochondrial fragmentation and apoptosis in retinal endothelial cells (13). Post-translational modifications can regulate protein-protein interaction, enzymatic activities and influence their cellular localization (39), and about 35% of all mitochondrial proteins are endogenously acetylated (40). Our results demonstrate that GTPase activity of Mfn2 is decreased in diabetes and its acetylation is increased, resulting in the fragmented mitochondria. Acetylation itself is a highly dynamic process, which is governed by balanced action between lysine acetyltransferases and deacetylases, and Sirt1 is one of the major deacetylases (41). Our results clearly demonstrate that Sirt1 activator ameliorates Mfn2 acetylation and mitochondrial fragmentation, induced by high glucose. In support, Sirt1 activator is shown to upregulate Mfn2 expression in gastric cancer cells (18). Moreover, Sirt1 activity is inhibited in diabetes and its expression is downregulated, and mice overexpressing *Sirt1* are protected from diabetes-induced retinal mitochondrial damage, vascular dysfunction and the development of histopathology characteristic of diabetic retinopathy (19).

To maintain mitochondrial quality control, damaged/defective mitochondria are removed by a conserved intracellular degradation mechanism, where the damaged/depolarized mitochondria are selectively sequestered into double-membraned autophagosomes, eventually fusing with lysosomes to generate single-membrane autolysosomes that mediate the degradation of the mitochondria (42, 43). Results presented here demonstrate that the formation of autophagosome and the mitochondrial levels of LC3B, a central protein which functions in the autophagosome biogenesis, are decreased in hyperglycemia. This is accompanied by poor entrapment of the damaged mitochondria inside the autolysosomes and overall decrease in the mitophagy flux. However, overexpression of *Mfn2*, or inhibition of its acetylation, ameliorates decrease in autophagosomes and the mitophagy flux, suggesting a critical role of Mfn2 in the removal of the damaged mitochondria. In support, mitophagy and mitochondrial dynamic are highly integrated at the functional level, and Mfn2 is considered to act as a linking protein that integrates mitochondrial network remodeling with mitophagy. It is also associated with the autophagosome formation and fusion of autophagosome-lysosome (42, 44). The effect of mitofusin on mitophagy is considered to be context-dependent, and its responsiveness can vary in different cell conditions; consistent with our results showing amelioration of hyperglycemia-induced decrease in autophagosome formation-mitophagy by *Mfn2* overexpression, downregulation of Mfn2 is associated with decrease in mitophagy in coronary heart disease (8). Mfn2 deficiency reduces autophagic flux, and during aging, it leads to the accumulation of damaged mitochondria and muscle atrophy (45, 46). Lysosomal impairment is also shown to depend on ROS, and the structure and function of lysosomes can be disrupted by mitochondrial dysfunction (47); ROS levels are elevated and mitochondria are dysfunctional in diabetic retinopathy. Furthermore, in diabetes, decreased mitophagy is associated with cardiomyopathy, kidney disease and impaired wound healing (48–50), and mitophagy in retinal pigment epithelial cells is decreased in high glucose conditions (51). Decreased levels of Mfn2, in addition to impairing mitochondrial dynamics and inhibiting removal of the damaged mitochondria, also increase cell apoptosis; in diabetic retinopathy apoptosis of capillary cells increased (13, 52, 53). Amelioration of high glucose-induced decrease in autopahagosome formation- mitophagy flux by resveratrol in retinal endothelial cells is further supported by others showing the role of Sirt1-Mfn2 axis in mitochondrial homeostasis (17, 18), and Sirt1 inhibition reducing Mfn2-mediated mitophagy in gastric cancer (18).

Alterations in mitochondrial membrane potential is considered as one of the initiator of their removal (54). Results presented here show that high glucose-induced alteration in mitochondrial membrane potential are ameliorated by inhibiting acetylation, or by overexpressing *Mfn2*, further strengthening the role of Mfn2 in the removal of the damaged mitochondria, generated during hyperglycemic insult.

Results obtained from retinal endothelial cells demonstrating decrease in the GTPase activity of Mfn2 and its acetylation in high glucose medium are strongly supported by similar findings from the *in vivo* model; GTPase activity of Mfn2 is decreased and its acetylation is increased in the retina from diabetic mice. Furthermore, *Sirt1* overexpressing mouse model, the model which is protected from the development of diabetic retinopathy and retinal mitochondrial damage (19), is also protected from diabetes-induced increase in Mfn2 acetylation and decrease in its GTPase activity. In addition, diabetes decreases autophagosome formation and mitophagy in the retinal mitochondria, and *Sirt1* overexpression ameliorates this, further strengthening the role of Mfn2 activation in the mitochondrial dynamic and removal of the damaged mitochondria in diabetic retinopathy.

We recognize that in a depolarized mitochondria, PINK1 accumulates on the outer mitochondrial membrane, leading to the phosphorylation of Mfn2. Phosphorylated Mfn2 serves as a receptor for inactive Parkin, activating ubiquitin ligase activity to initiate the removal process (55), however, this is beyond the focus of this study. Furthermore, we cannot rule out the possibility that the impairment in the Mfn2-Parkin-ubiquitination axis could also be contributing in the poor removal of the damaged mitochondria in diabetic retinopathy.

**Figure 9:**
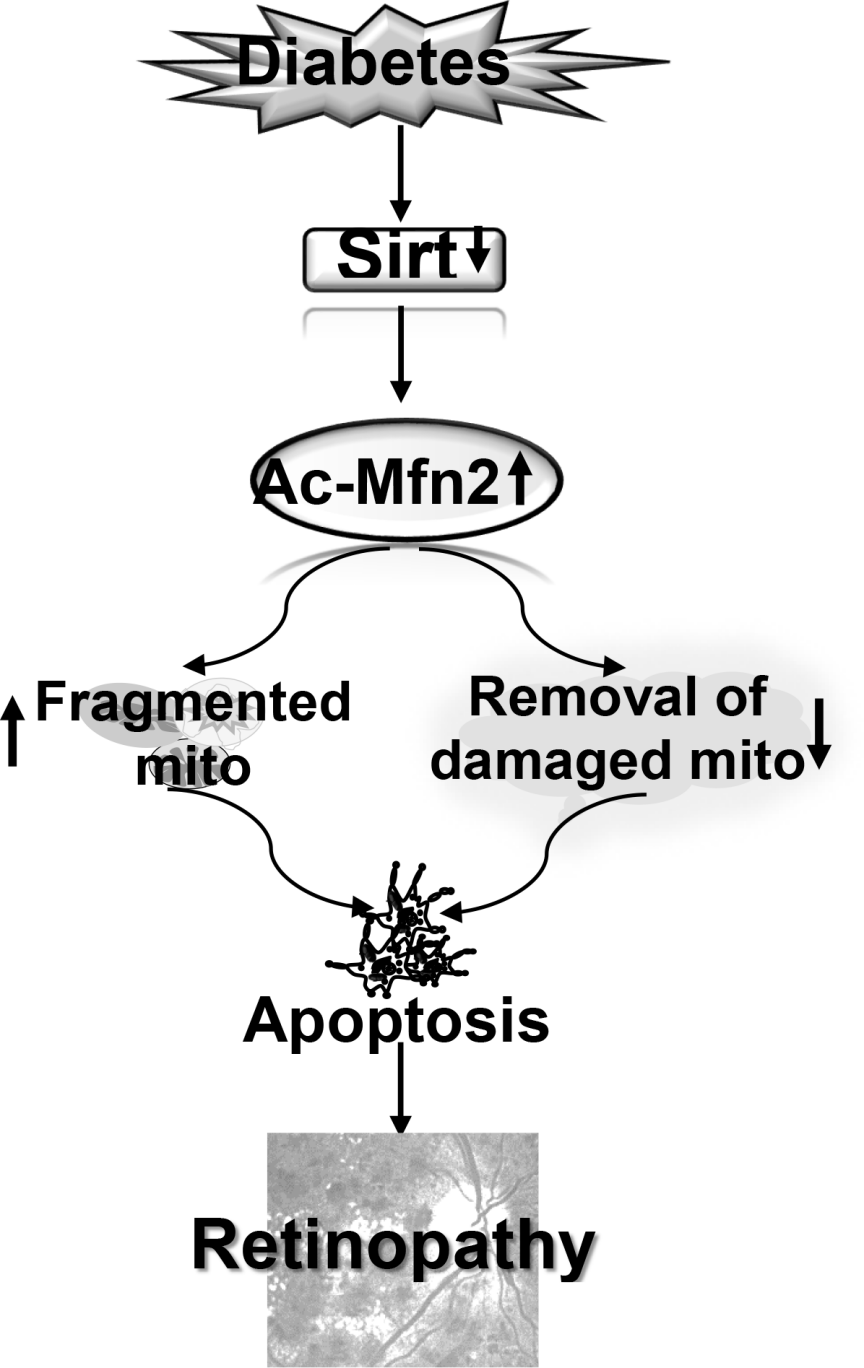
Model showing regulation of Mfn2 and its dual role in diabetic retinopathy. Inhibition of Sirt1 in diabetes results in hyperacetylation of Mfn2 (Ac-Mfn2), which inhibits its GTPase activity. This leads to fragmentation of the mitochondria, and also in the impaired removal of the damaged mitochondria. Continued accumulation of the dysfunctional mitochondria accelerates capillary cell apoptosis, which ultimately leads to the development of diabetic retinopathy.

In summary, in diabetic retinopathy Mfn2 has an important role in both mitochondrial dynamics and removal of the damaged mitochondria. Activation of DNA methylation machinery in diabetes downregulates retinal *Mfn2* gene transcripts (13), and here we show that Sirt1 inhibition leaves it hyperacetylated with reduction in its GTPase activity. This imbalances mitochondrial fusion-fission, and results in the accumulation of the fragmented-dysfunctional mitochondria. Acetylated Mfn2 further worsens mitochondrial homeostasis by impairing removal of the damaged mitochondria, and the damaged mitochondria continue to accumulate and produce free radicals (**Figure 9**). Thus, maintenance of Mfn2 activity, by targeting its acetylation, in addition to preventing impairments in mitochondrial dynamics, has potential to improve clearance of the damaged- fragmented mitochondria, which could inhibit the development/progression of diabetic retinopathy.

## Data availability statement

The original contributions presented in the study are included in the article/supplementary material. Further inquiries can be directed to the corresponding author.

## Ethics statement

The animal study was reviewed and approved by Wayne State University’s Animal Care and Use Committee.

## Author contributions

Conceptualization, RK. Data curation, KA. FACs data curation & analysis, JK. Funding acquisition, RK. Project administration, RK. Writing- original draft, RK. Review and editing, KA, JK and RK. All authors contributed to the article and approved the submitted version.
