# Correction: Impaired mitochondrial dynamics and removal of the damaged mitochondria in diabetic retinopathy

**DOI:** 10.3389/fendo.2026.1793182

**Published:** 2026-04-24

**Authors:** Kumari Alka, Jay Kumar, Renu A. Kowluru

**Affiliations:** Ophthalmology, Visual and Anatomical Sciences, Wayne State University, Detroit, MI, United States

**Keywords:** diabetic retinopathy, mitochondria, mitofusin, mitophagy, mitochondrial dynamics, retina

There was a mistake in **Figure 2** as published. While compiling original **Figure 2a**, inadvertently wrong images with overlapping field of view were used for NG and HG groups. Increased acetylation of Mfn2 in high glucose, as shown in **Figure 2a**, was further supported by increased fluorescence of acetyl lysine and Mfn2:acetyl lysine Pearson correlation coefficient, and by the co-immunoprecipitation data (original **Figures 2b–e**).

**Figure 2:**
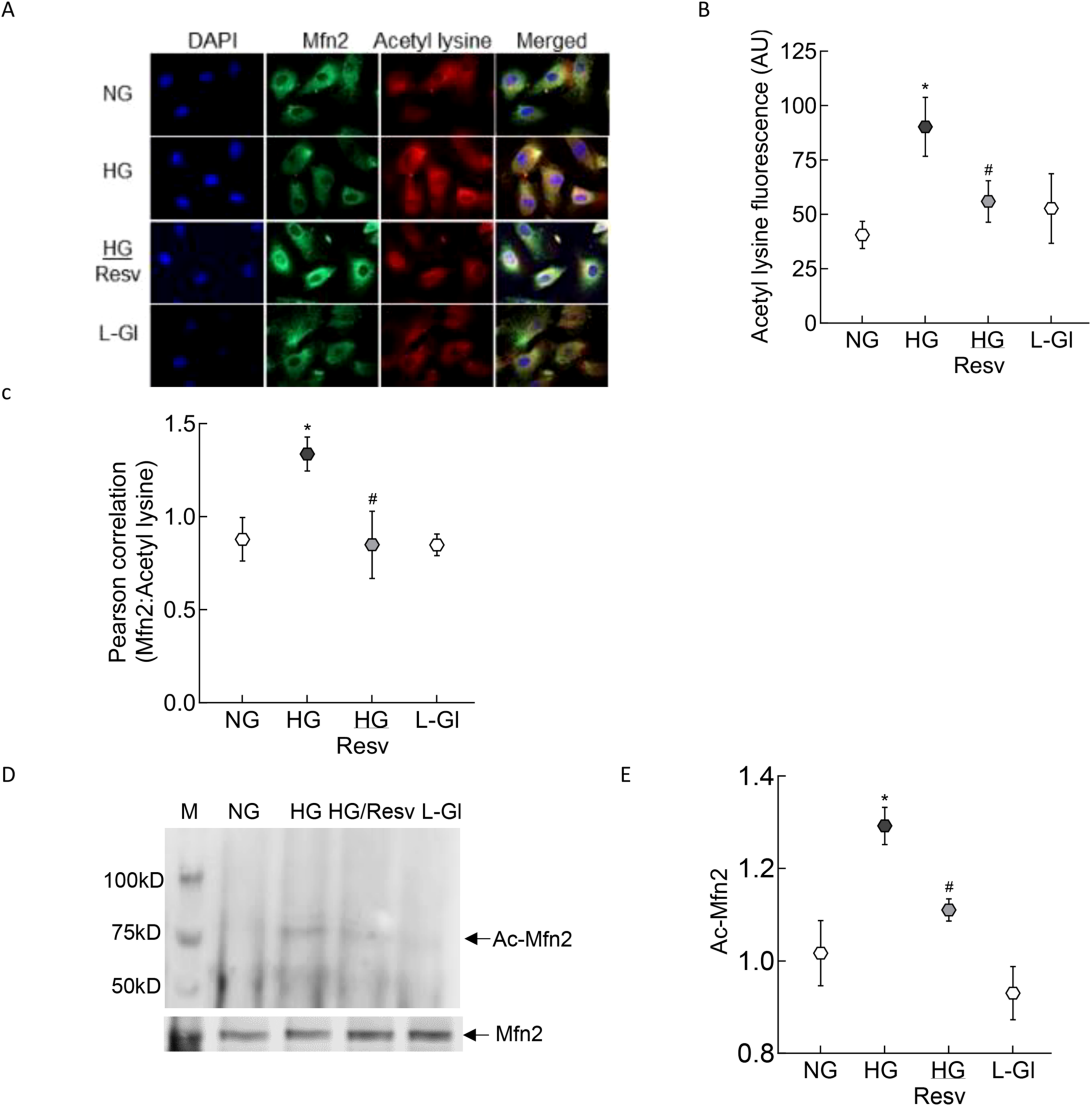
Acetylation of Mfn2. **(A)** Representative immunofluorescence image showing staining of Mfn2 (Alexa Fluor-488 conjugated secondary antibody, green) and acetyl lysine (Texas red-conjugated secondary antibody). Cells were imaged under an ApoTome microscope with a 20X objective. **(B)** Fluorescence intensity of acetyl lysine was quantified using Zeiss software module and the data are expressed as arbitrary units (AU). **(C)** Pearson correlation coefficient between acetyl lysine and Mfn2 was calculated using Zeiss software module. **(D)** Representative image of Mfn2 western blot in acetyl lysine immunoprecipitated HRECs. **(E)** Intensity of the acetylated Mfn2 (Ac-Mfn2) was quantified by image J software. The values are represented as mean ± SD. NG= 5mM D-glucose, HG= 20mM D-glucose, HG/Resv = cells incubated in 20mM D-glucose in the presence of resveratrol; L-Gl= 20mM L-glucose.*p<0.05 vs NG and #p<0.05 vs HG.

The corrected **Figure 2** appears below.

The original version of this article has been updated.

